# PARS: Privacy-Aware Reward System for Mobile Crowdsensing Systems

**DOI:** 10.3390/s21217045

**Published:** 2021-10-24

**Authors:** Zhong Zhang, Dae Hyun Yum, Minho Shin

**Affiliations:** 1 Department of Computer Engineering, Myongji University, Yongin 17058, Korea; zhangzhong219017@hotmail.com; 2 Department of Information and Communication Engineering, Myongji University, Yongin 17058, Korea; dhyum@mju.ac.kr

**Keywords:** mobile crowdsensing, privacy-preserving, blind signature, incentive, aggregation

## Abstract

Crowdsensing systems have been developed for wide-area sensing tasks because humancarried smartphones are prevailing and becoming capable. To encourage more people to participate in sensing tasks, various incentive mechanisms were proposed. However, participating in sensing tasks and getting rewards can inherently risk the users’ privacy and discourage their participation. In particular, the rewarding process can expose the participants’ sensor data and possibly link sensitive data to their identities. In this work, we propose a privacy-preserving reward system in crowdsensing using the blind signature. The proposed scheme protects the participants’ privacy by decoupling contributions and rewarding claims. Our experiment results show that the proposed mechanism is feasible and efficient.

## 1. Introduction

Since the advent of smartphones, mobile devices began prevailing in human lives and they became essential to our daily lives as well as professional activities. The sales of the mobile devices are expected to keep increasing in the future [1,2,3]. These mobile devices are equipped with various sensors (such as accelerometer, gyroscope, GPS, microphone, and camera). Sensors in a mobile device can collect different kinds of information about the users, their contextual situation, and surrounding environment. Crowdsensing is a category of applications leveraging the sensing capabilities of each mobile device and its perpetual connectivity. A crowdsensing system can collect sensor data from a number of mobile devices and use the data to measure and map the phenomena of common interest. Compared to the traditional methods for collecting sensor data, such as installing sensors at the field and deploying a sensor network, the crowdsensing system can collect data more efficiently and economically [4,5]. Researchers developed many crowdsensing systems; they can monitor the environment [6,7,8,9,10], take care of the users’ health [11,12,13,14], and monitor and improve the traffic conditions [15,16,17,18].

Despite the convenience and low cost, crowdsensing systems need a number of participants to get enough data. The privacy of the device owners in a crowdsensing system can be at risk. The sensor data collected from an individual’s device can reveal sensitive information about the user. Certain data can directly reveal the personal information of the users such as location [15,16,17,18] or health status [11,12,13,14]. Indirectly related data can be even analyzed to infer private information. For instance, the accelerometer data, which is hardly a sensitive data by itself, can be used to classify the user’s activities [19]; the GPS traces can be used to identify the user’s identity [20]; and the motion sensor data can be used to infer the user’s password [21,22].

However, the privacy issues may discourage the users from use the crowdsensing systems. Privacy-preserving crowdsensing systems have been proposed to address the reluctance of participation by concerned users [23]. PoolView [24] perturbs data before submission to preserve privacy. PEPSI [25] encrypts the sensor reports to prevent linking between reports. In Prisense [26], reports are forwarded among users before being sent to the server in order to hide the origin of the data. Anonysense [27] provides user anonymity using group signature to prevent the linking between multiple tasks and between multiple reports. Incognisense [28] and ARTSense [29] provide user anonymity using blind signature to prevent the linking between the multiple reports from the same user.

Protecting privacy, however, is not enough to incentivize participation. Several efforts have been made to propose incentive mechanisms to attract the users. For example, the authors of [30,31,32] proposed auction based incentive mechanisms. In [33], the participants can earn credits in exchange of their sensor data. Micro-payments for shared data can be effective as well [34,35,36]. Game theory was also applied to enhance data quality with rewards to participants [37,38].

Unfortunately, an incentive mechanism may expose the users to privacy risks. A typical rewarding process can reveal the identity of the recipients. First, the users have to prove their contribution to data collection, which may reveal who they are, when and where the data was collected, and even what was the data [39]. Second, after verifying contribution, the rewarding procedure may also reveal the beneficiary’s identity depending on the form of rewards (e.g., account credits, bank transfer, or gift delivery). The authors of [40] proposed an auction-based incentive mechanism with encrypted bidding. Although encryption can protect unauthorized access to the incentive mechanism, the honest-but-curious rewarding server can still access the contributor’s information and can try to link the contribution information, which can reveal personal information, to the beneficiary such as IP address, account id, or bank account number. To hide the identity of the beneficiary, a collaboration between participants was proposed [41,42]. In [41], the data from different users will be aggregated before being sent to the server. In [42], the system provides user anonymity by distributing a task to a group of participants and using anonymously exchangeable currency E-cent. Group signatures and ring signatures were also applied for user anonymity in the process of rewarding in [35,43].

Even if the beneficiary remains anonymous to the reward server (for example, the connection is anonymous and the reward is given anonymously), the attacker can collude with the reward server and the data collection server, one of which knows the identity of the user. Privacy-preserving incentive mechanisms such as those in [35,43] are not resilient to colluding attacks because of the trusted entity in the system. In both systems, the trusted entity is not supposed to be malicious. If the attacker can collude with the trusted entity and other entities, the users’ identity might be revealed. In [44,45], the researchers used blind signature, partially blind signature, and Merkle tree to prevent colluding attacks. They strategies can prevent the server from learning the token information during credit deposition by blindly signed tokens. However, the server manages the credit account information and updates the user’s reward. The updated rewards can be used to figure out the related contributions, so infer the user’s task information.

In this paper, we aim to design a privacy-preserving contribution rewards system to incentivize the mobile crowdsensing systems. In particular, we focus on two kinds of linking attacks: linking a contribution to a reward claim and linking multiple contributions to the same user. We assume that the data collection server and the reward server can collude, or they can be even integrated into one. We also focus on the efficiency of the rewarding process. The followings are the contribution of this paper.

- We identified two kinds of linking attacks against user privacy in incentivized crowdsensing systems.
- We proposed a novel privacy-preserving reward mechanism for crowdsensing.
- We formally proved the security of the mechanism.
- We verified the feasibility of the system by the implementation of the mechanism on a real mobile device.

This paper consists of seven sections. After the introduction in the first section, we formalize the system model, threat model, and security goals in Section 2. Then, we describe our approach in Section 3. In Section 4, the evaluation of our approach and the experiment results are given. Some further discussions are in Section 5. Section 6 explains related works. At the end is the conclusion in Section 7.

## 2. Problem Formulation

In this section, we describe the system model and security properties that the system wants to achieve.

### 2.1. System Model

Our system model is generic so that it can represent different types of architectures and applications. For example, we consider the case of centralized crowdsensing system where one cloud server tasks a vast number of smartphones to report their sensor data, collect them, and analyze the data. Meanwhile, the participating users will contact to a separate *Reward Service* to get the rewards for their contributions. However, our mechanism also works when the data collector and *Reward Service* are on the same server or run by the same entity. The scope of this work is not limited to a typical crowdsensing system. Our system model also includes local tasking systems such as sensor sharing [46,47]. In these systems, a mobile device can task other devices in its vicinity through a local connection such as WiFi or Bluetooth. The helping devices can perform data collection or computation on behalf of the requesting device and report back the results. The contributor will later redeem their rewards from the *Reward Service* which has a contract with the tasking users. The system model also does not make any assumption about the communication medium between actors. Such flexibility even allows our privacy mechanism to improve daily shopping experiences in the form of privacy-aware coupon. For the sake of exposition, we provide a formal definition about the system model as follows.

*Master* (M) is an entity that creates a task on behalf of its owner or any external data consumer, disseminates the task, and collects the reports for the task. *Helper* (H) is an entity that receives a task from the *Master*, performs the task, and reports the results back to the *Master*. *Reward Service* (RS) is the entity that verifies the contribution of an *Helper* and issues rewards to the *Helper* accordingly. *Certificate Authority* (CA) is an authorized entity that issues certificates to entities and checks the validity of certificates if requested. The distinction between CA and RS is only logical separation, and CA and RS can be implemented in the same server.

**Figure 1:**
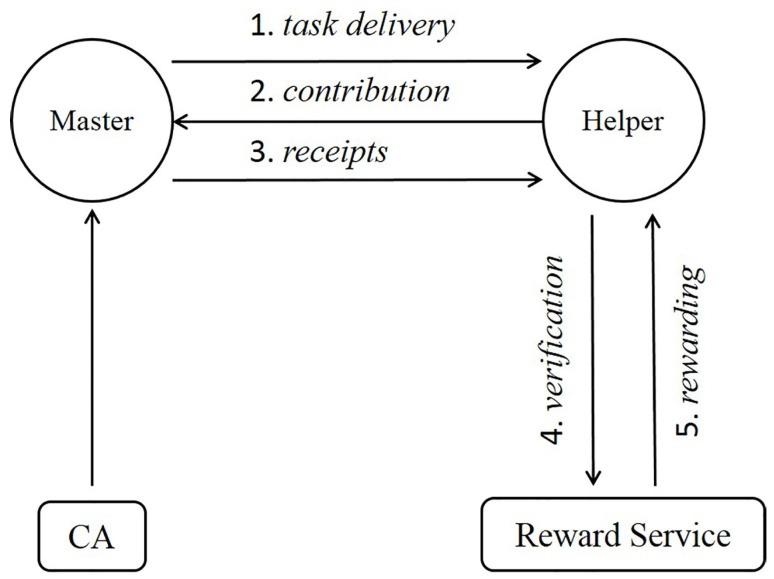
System model.

The system operates as illustrated in Figure 1. At step 1, the *Master* creates a task (*t*) and sends it to the *Helper*. The task can originate from the *Master* itself, but it can also come from a data consumer external to the *Master* when the *Master* is a query distribution service [27,40,43]. The task may be written in a task-definition language [35], a generic script language, or an executable file. A task can be delivered to potential *Helpers* in many different ways. Crowdsensing system often has its own mechanism for task delivery but typically disseminates the task only to qualified devices (registered and meets the task pre-condition), which then voluntarily decides to run the task, possibly motivated by the incentive mechanism in place. In local settings, where *Master* and *Helper* are in a direct communication range, the *Master* will broadcast the task on a wireless medium hoping to get accepted by nearby devices [46,47].

Once a *Helper* decided to perform the task, it will send back the result of the task execution, whether raw sensor data, processed sensor data, or computation results (Step 2). Upon the reception of the contribution from *Helper*, *Master* issues a receipt of the contribution (step 3), as a credential to prove the *Helper*’s effort, to be rendered by the *Helper* to RS later on.

The rewarding procedure consists of the verification (Step 4) and rewarding step (Step 5). At Step 4, *Helper* and RS run a verification protocol to determine if the *Helper* has made a valid contribution and the contribution has not been redeemed before. For the verification, the *Helper* may use the receipt provided by the *Master* or some other credential derived from the receipt. Once verified, the RS will issue a reward to the *Helper*. The reward can be any form of digital credit such as e-coupon, voucher, or cryptocurrency, and our mechanism does not depend on the format of the reward.

### 2.2. Threat Model

In this section, we describe what kind of threats we are concerned about and who can be potential adversaries to impose those threats.

We are mainly concerned about the privacy of the *Helper*’s user, who is reluctant to participate if their contribution to the *Master* or the rewarding process can reveal some sensitive information. A piece of typical sensitive information is location. Most sensing tasks, if not all, require the location information to be reported along with sensor data. When *Master* and *Helper* are local, the *Master* certainly knows that the *Helper* is near itself. Other information submitted to the *Master* can also directly reveal sensitive information of the user, or can be used to infer personal information. What information will be revealed to the *Master* depends on the type of the task and the protocol design.

Therefore, there should be an implicit or explicit agreement between the *Helper* and *Master* that the *Helper* is willing to share some personal information with the *Master* as part of the contribution, and the *Master* would respect the *Helper*’s privacy. There are still remaining issues for how to prevent the *Master* from learning more information than what it was agreed beforehand. In this work, we assume that the privacy concern against the *Master* with respect to the data itself is already resolved [27] and further protection is out of the scope.

On the other hand, the *Master* can be concerned whether the contribution data from the *Helper* is trustworthy or not. A *Helper* may attempt to make counterfeit contribution by providing the *Master* with false reports in response to the task given by the *Master*. For example, when the *Master* asked to report the current temperature, the *Helper* may report with fake temperature values. This study does not address this issue. Studies on how to ensure the integrity of sensing reports in crowdsensing systems can be found in [48,49].

The *Reward Service* (RS) is a third party and the *Helper* has no reason to trust the RS. The *Helper* expects for the RS to provide rewards that it deserves. On the other hand, the RS has to verify the contribution claimed by the *Helper* before handing out a reward. For the verification, the *Helper* needs to provide some evidence that can convince the RS of its contribution. However, once the RS learns about the contribution made by the *Helper*, the related contributions may help the RS to infer further information about the *Helper* such as locations and device capabilities. To that end, the RS may collude with *Masters* to ease the attack. (**T1**)

The *Helper* is also concerned about whether the RS will provide rewards that it deserves, and whether the *Master* will cooperate in that regard. For example, the RS can just deny the fact that the *Helper* actually contributed to a task, or the *Master* may refuse to provide information necessary for the RS to verify the contribution. They may have motivation to do that, because the reward could cost the *Master* (if the task was issued by the *Master*) or the RS (if the RS issued the task). It is challenging for the *Helper* to ensure that the RS and the *Master* cannot repudiate the contribution. (**T2** and **T3**)

On the flip side, the RS and the *Master* may be concerned about a malicious *Helper* who claims a reward without making any contribution. For example, the *Helper* may bring a forged evidence of contribution to get an undeserved reward, or may repeatedly claim rewards for the same contribution. Therefore, the RS and *Master* want to make sure that there is a mechanism to mathematically disprove the validity of illegitimate claims of rewards. (**T4** and **T5**)

In summary, the attacker, who can be either *Helper*, *Master*, or RS, can pose the following threats against the *Helper*’s privacy or resource, or the *Master* and RS’s financial asset.

- **T1**: The RS may attempt to learn about the *Helper* more than necessary for rewarding.
- **T2**: *Master* may repudiate the *Helper*’s valid contribution.
- **T3**: RS may repudiate the *Helper*’s valid contribution.
- **T4**: *Helper* may claim rewards without contribution.
- **T5**: *Helper* may claim multiple rewards out of a single contribution.

### 2.3. Security Assumptions

For the system to work, we need some baseline assumptions between entities. For example, the *Helper* assumes that a legitimate *Master* will issue valid proofs for its contribution (**A1**). Furthermore, RS trusts the *Master* to issue the proof only when needed (**A2**). To authenticate the *Master*, we assume that there is a *Certificate Authority* (CA) that issues the *Master*’s certificate that can be verified by others (**A3**).

We assume that the RS may collude with the *Master* to compromise the *Helper*’s privacy (**A4**). The *Master* may provide necessary information to RS so that the RS can learn about the *Helper*’s contribution.

We assume that the *Master* receives contributions from multiple *Helpers* (**A5**). Without this assumption, it is obvious for the RS to identify the task and contribution of an *Helper* by colluding with that *Master*. If there are multiple *Helpers* that made contributions to the *Master*, the *Master* cannot identify which contribution a particular proof was linked to without a clear association between the contribution and the proof.

We make the following security assumptions:

- **A1**: The *Helper* trusts the *Master* to issue a valid proof for the contribution.
- **A2**: The RS trusts the *Master* to issue a valid proof only to a valid contribution
- **A3**: CA is a correctly functioning *Certificate Authority*.
- **A4**: RS and *Master* may collude to compromise the *Helper*’s privacy.
- **A5**: *Master* interacts with many *Helpers*.

### 2.4. Security Goals

Based on the threat model and security assumptions, we aim to achieve the following security goals:

- **G1: Privacy (against T1)** Rewarding process reveals no information about the contribution except the identity of the *Master*.
- **G2: Non-repudiation (against T2, T3)***Master* and RS cannot repudiate a claim with valid contributions.
- **G3: Accountability-1 (against T4)**RS can detect a false claim without actual contribution.
- **G4: Accountability-2 (against T5)**RS can detect multiple claims for the same contribution.

In order to achieve the security goals, we derive the security properties for the reward system to hold.

To achieve the goal **G1,** we need a security property such that no proof can be linked to the contribution. That is, the RS should be able to verify that the *Helper*, presenting a proof of the contribution, indeed made a contribution to the *Master* without knowing which contribution the *Helper* made. This **proof-to-contribution unlinkability** (**P1**) makes the reward process non-trivial for RS as it has to verify the *Helper*’s contribution without knowing the contribution. Even if proof-to-contribution is unlinkable, RS can identify two different proofs coming from the same *Helper*. This will help the attacker to identify the contributions from the same *Helper* and infer the *Helper*’s identity. Therefore, we need **contribution-to-contribution unlinkability** (**P2**).

To achieve **G2**, the *Helper* should be able to mathematically prove that it made a contribution to the *Master* and the *Master* issued a proof of it. Suppose the RS has a verification function. For the RS to be able to repudiate, it should be possible that the verification fails for a valid contribution. Therefore, to prevent repudiation of RS, it should be mathematically proven that all proofs that passes the verification have a valid contribution (**P3**).

To achieve **G3** and **G4**, the proof unforgeability should be provided. When RS receives proof, a mathematical technique should be used for RS to verify whether the proof corresponds to a valid contribution. When RS receives a double-spent proof, a mathematical technique should be used for RS to verify whether the proof corresponds to an already claimed contribution. In other words, a mathematical technique should be used to make sure that a valid proof corresponds only to one valid contribution. (See **P4**)

We summarize the security properties as follows:

- **P1: Proof-to-contribution unlinkability (G1)**. Difficult to identify the contribution that corresponds to a specific proof.
- **P2: Contribution-to-contribution unlinkability (G1)**. Difficult to link the contributions that come from the same *Helper*.
- **P3: Non-repudiation (G2)**. Given a proof, one can mathematically prove that there exists a corresponding contribution.
- **P4: Proof unforgeability (G3, G4)**. Given an illegitimate proof (invalid or double-spent), one can mathematically disprove that there is no corresponding contribution that has not been claimed before.

## 3. Privacy-Aware Reward System

A reward scheme can be defined by four algorithms as follows.

**Definition** **1** (Reward Scheme).

*A reward scheme consists of four algorithms (Kg,Helper,Master,Vrfy) such that:*

- *The key generation algorithm Kg is a probabilistic polynomial time algorithm, which takes as input a security parameter k (encoded as $1^{k}$) and outputs a pair of keys (sk,vk). These are called the receipt-issuing key and the receipt-verification key, respectively. We write the execution of the key generation algorithm as $\left(\mathrm{sk},\mathrm{vk}\right)\leftarrow \mathrm{Kg}\left(1^{k}\right)$.*
- *Master and Helper are interactive probabilistic Turing machines that run in polynomial time. As inputs, Master is given (sk,vk) and Helper is given a serial number s and vk. After the joint execution of Master and Helper, the Helper algorithm outputs a receipt σ (i.e., σ is only known to Helper). We write the receipt-issuing process as σ←〈Master(sk,vk),Helper(s,vk)〉. If the joint execution is incomplete or one aborts, then σ is set as* ⊥ *(which is never a valid receipt).*
- *The verification algorithm Vrfy is a deterministic polynomial time algorithm which takes as input a verification key vk, a serial number s, a receipt σ, and a list L that contains all expired serial numbers and outputs either valid or invalid. We write this as valid/invalid←Vrfy(vk,s,σ,L).*

*It is required that for all (sk,vk) output by $\mathrm{Kg}(1^{k})$, if s∉L and σ←〈Master(sk,vk), Helper(s,vk)〉, it holds that Vrfy(vk, s, σ,L)=valid and if s∈L, it holds that Vrfy(vk,s,σ,L)=invalid for any σ.*

We will design our reward scheme based on a Gap Diffie–Hellman (GDH) group where the Computational Diffie-Hellman (CHD) problem is hard, but the Decisional Diffie–Hellman (DDH) problem is easy [50]. Although general GDH groups are sufficient for constructing our scheme, our description uses GDH groups with a bilinear map, which enables us to aggregate multiple receipts into a single receipt of a constant length.

Let G and $G_{T}$ be multiplicative cyclic groups of prime order *p* where the group operation on G and $G_{T}$ can be computed efficiently. Let *g* be a generator of G and $e:G\times G\to G_{T}$ be an efficiently-computable bilinear map with the following properties.

1. Bilinear: for all u,v∈G and a,b∈Z, $e\left(u^{a},v^{b}\right)=e{\left(u,v\right)}^{ab}$.
2. Non-degenerate: e(g,g)≠1.

These properties implies that for any $u_{1},u_{2}\in G$, $e\left(u_{1}u_{2},v\right)=e\left(u_{1},v\right)\cdot e\left(u_{2},v\right)$. The group G with a bilinear map is called a bilinear group. Joux and Nguyen [50] showed that an efficiently-computable bilinear map *e* provides an algorithm for solving the DDH problem: for a tuple $\left(g,g^{a},g^{b},g^{c}\right)$ where a,b,c∈Z, we have c=abmodp if and only if $e\left(g^{a},g^{b}\right)=e\left(g,g^{c}\right)$.

Our reward scheme is based on the GDH blind signature scheme of Boldyreva [51] that is an extension of the GDH signature scheme of Boneh et al. [52,53]. The security of the GDH blind signature scheme assumes that the chosen-target CDH problem is hard.

**Definition** **2** (The Chosen-Target CDH Problem and Assumption). Let g be a generator of a cyclic group G of prime order p. Let x be a random element of $Z_{p}^{*}$ and let $y=g^{x}$. The adversary B is given (p,g,y) and has access to the target oracle $T_{G}$ that returns random elements z∈G and the exponentiation oracle ${𝛆}_{x}$ that takes as input an element α∈G and returns $\alpha^{x}$. Let $q_{T}$ (resp. $q_{𝛆}$) be the number of queries B made to the target (resp. exponentiation) oracle such that $q_{T}>q_{𝛆}$. Let $z_{i}\in G$ for $1\leq i\leq q_{T}$ be the values returned by $T_{G}$. The advantage of the adversary attacking the chosen-target CDH problem ${\mathrm{Adv}}_{G}^{\mathrm{ct}-\mathrm{cdh}}\left(B\right)$ is defined as the probability of B to output a set V of q𝛆+1 pairs $\left(\left(v_{1},j_{1}\right),\left(v_{2},j_{2}\right),\ldots ,\left(v_{q𝛆+1},j_{q𝛆+1}\right)\right)$, where for 1≤i≤q𝛆+1, all $v_{i}$ are distinct, $1\leq j_{i}\leq q_{T}$, and $v_{i}=z_{j_{i}}^{x}$. The chosen-target CDH assumption states that the advantage ${\mathrm{Adv}}_{G}^{\mathrm{ct}-\mathrm{cdh}}\left(B\right)$ of any probabilistic polynomial time adversary B is negligible.

We propose a reward scheme that is unforgeable, contribution unlinkable, and proof-contribution unlinkable under the chosen-target CDH assumption in the random oracle model. Consider multiplicative cyclic groups G and $G_{T}$ of prime order *p* with a generator *g* of G and a bilinear map $e:G\times G\to G_{T}$, where the bit length of *p* is determined by the security parameter *k* (i.e., |p|=k). Let S be a serial number space whose size is super-polynomial (e.g., ${\left\{0,1\right\}}^{k}$). The scheme employs a full-domain hash function $H:{\left\{0,1\right\}}^{*}\to G$ viewed as a random oracle [54]. The proof of possession of the receipt-issuing key can be performed by generating a BLS signature [53] on a random challenge. Let C be the set of random challenges. It is required that the challenge space C and the serial number space S are disjoint (i.e., C∩S=∅), which guarantees that a proof of possession cannot be misused as a receipt.

Our reward scheme is as follows.

- **Key generation:** The key generation algorithm Kg selects a random number $x\overset{R}{\leftarrow }Z_{p}$ and computes $y\leftarrow g^{x}$ where $\overset{R}{\leftarrow }$ denotes a uniformly random choice. The receipt-issuing key sk is *x* and the verification key vk is y∈G.
- **PoP generation:** The verifier (e.g., Helper) chooses a random challenge $ch\overset{R}{\leftarrow }C$ and sends ch to Master. After receiving ch, the Master checks ch∈C, computes $\lambda \leftarrow H{\left(ch\right)}^{x}$, and sends λ to the verifier.
- **PoP verification:** The verifier checks the validity of λ; if e(λ,g)=e(H(ch),y) holds, valid is returned and otherwise, invalid is returned.
- **Receipt-issuing process:** The Helper algorithm selects a random serial number $s\overset{R}{\leftarrow }S$ and a random number $r\overset{R}{\leftarrow }Z_{p}$, computes $h\leftarrow g^{r}H\left(s\right)$, and sends *h* to Master. After receiving *h*, the Master computes $\psi \leftarrow h^{x}$ and sends ψ to Helper. Finally, Helper computes the receipt $\sigma \leftarrow y^{-r}\psi$ for the serial number *s*.
- **Verification:** The verification algorithm Vrfy first checks the freshness of the serial number *s*; if s∉S or s∈L, invalid is returned. Vrfy then checks the authenticity of the receipt σ; if e(σ,g)=e(H(s),y) holds, valid is returned and otherwise, invalid is returned.

Each algorithm can also validate its input and output. For example, Master can check that *h* is an element of G (i.e., h∈G) and Helper can check that σ is the valid receipt for *s* by running Vrfy(y,s,σ,∅). If the key generation and the receipt-issuing process are executed correctly, we have

(1) $$\begin{array}{cc} e(\sigma ,g) & =e\left(y^{-r}\psi ,g\right)=e\left(y^{-r}h^{x},g\right)=e\left(y^{-r}{\left\{g^{r}H\left(s\right)\right\}}^{x},g\right)\\ \; & =e\left(y^{-r}\left\{y^{r}H{\left(s\right)}^{x}\right\},g\right)=e\left(H{\left(s\right)}^{x},g\right)=e\left(H\left(s\right),g^{x}\right)\\ \; & =e(H(s),y) \end{array}$$

which shows that σ is the valid receipt for *s*.

**Figure 2:**
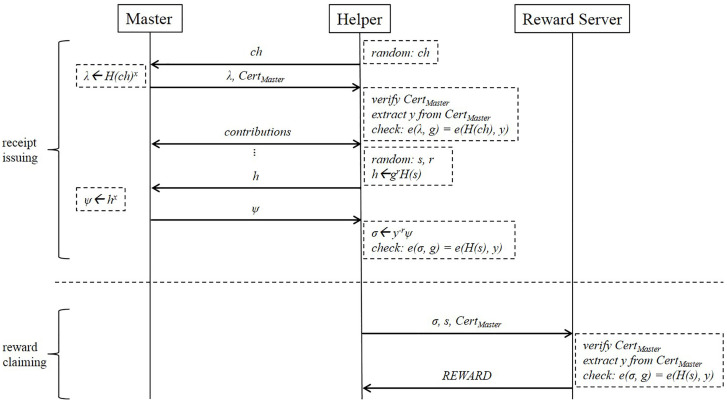
Protocol of reward scheme.

As shown in Figure 2, our reward scheme is used in the following way. A user, who acts as a *Master*, runs $\mathrm{Kg}(1^{k})$ to obtain (x,y). The receipt-issuing key *x* is kept secret and the verification key *y* is certified by a CA in the form of digital certificate. The certificate issuing procedure is out of scope, but, for example, the *Master* will send a certificate signing request (CSR) message to the CA, and the CA will issue a certificate $Cert_{Master}$ in return. The *Helper* can verify the *Master*’s identity by verifying the signature of CA on the certificate in order to prevent a malicious *Master* from tasking the *Helper* without rewards. When another user, who acts as a *Helper*, wants to get a receipt after contributing to the *Master*’s task, σ←〈Master(x,y),Helper(s,y)〉 is executed jointly by *Master* running Master(x,y) and *Helper* running Helper(s,y). At the first time of the communication between the *Master* and the *Helper*, the *Helper* needs to authenticate the *Master* with a challenge ch. The *Master* signs on ch using *x*, and sends $\lambda \leftarrow H{\left(ch\right)}^{x}$ and $Cert_{Master}$ to the *Helper*. Note that *y* can be extracted from $Cert_{Master}$. The fresh serial number *s* is usually chosen by *Helper* uniformly at random during the receipt-issuing process (i.e., *s* is essentially a random nonce) and the receipt σ is issued for the serial number *s*. To exchange a receipt for a reward, *Helper* sends a reward-requesting message $\left(s,\sigma ,Cert_{Master}\right)$ to the *Reward Serivce* RS who can verify the authenticity of (s,σ) by checking whether $\mathrm{Vrfy}\left(y,s,\sigma ,L\right)\overset{?}{=}\mathrm{valid}$ of not. If σ is a valid receipt for an unused serial number *s*, RS sends a reward to *Helper* and adds *s* to the expiration list *L*.

Receipt $\sigma =y^{r}\psi =H{\left(s\right)}^{x}$ is the GDH signature of Boneh et al. [52,53]. A nice property of the GDH signature is that multiple signatures by distinct entities on distinct messages can be aggregated into a single signature [55]. Suppose *Master* $U_{i}$ for 1≤i≤n has receipt-issuing key $x_{i}\in Z_{p}$ and verification key $y_{i}=g^{x_{i}}\in G$. The aggregate of *n* receipts $\sigma_{1},\sigma_{2},\ldots ,\sigma_{n}$ for $\sigma_{i}=H{\left(s_{i}\right)}^{x_{i}}$ can be computed as $\sigma \leftarrow \sigma_{1}\sigma_{2}\cdots \sigma_{n}\in G$ whose authenticity can be verified by $e\left(\sigma ,g\right)=\prod_{i=1}^{n}e\left(H\left(s_{i}\right),y_{i}\right)$.

**Figure 3:**
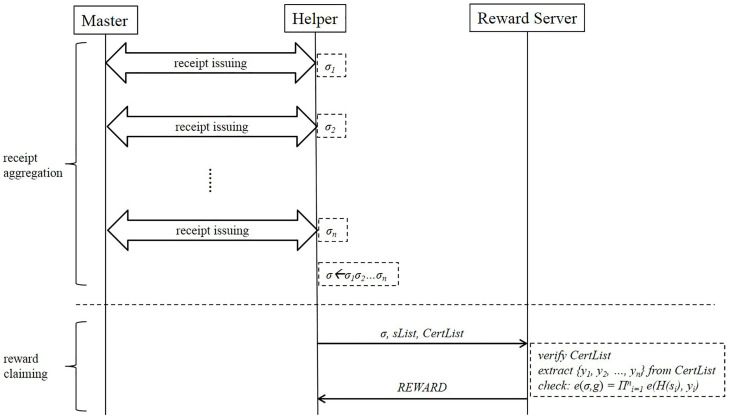
Protocol of reward scheme with aggregation.

As shown in Figure 3, when the *Helper* chooses to aggregate the receipts, the protocol for the receipt issuing is the same as in Figure 2. To aggregate the receipts, the *Helper* makes $\sigma \leftarrow \sigma_{1}\sigma_{2}\cdots \sigma_{n}$, and stores the corresponding serial numbers and certificates in lists. To get the reward from the RS, the *Helper* needs to send the aggregated σ, sList, and CertList to the RS, where $sList=(s_{1},s_{2},...,s_{n})$ and $CertList=(Cert_{1},Cert_{2},...,Cert_{n})$.

Instead of choosing a serial number randomly for each run of Helper, serial numbers can be generated deterministically by using a pseudorandom function $F_{\kappa }:S\to S$ with key κ. A *Helper* with an additional secret key κ can generate serial numbers by $s_{i}=F_{\kappa }\left(i\right)$, which is indistinguishable from random selection of serial numbers. By adopting aggregate receipts and pseudorandom function $F_{\kappa }$, the *Helper* can store $\left(\beta ,\gamma ,\sigma_{\beta \gamma }\right)$ in place of $\left(\left(s_{\beta },\sigma_{\beta }\right),\left(s_{\beta +1},\sigma_{\beta +1}\right),\ldots ,\left(s_{\gamma },\sigma_{\gamma }\right)\right)$ where $\sigma_{ij}=\sigma_{i}\sigma_{i+1}\cdots \sigma_{j}$ and $s_{i}=F_{\kappa }\left(i\right)$.

## 4. Results

### 4.1. Security Definition

We formally define the security properties P1, P2, and P4 as follows.

**Definition** **3** (Proof-to-Contribution Unlinkability). *A reward scheme R=(Kg,Helper,Master,Vrfy) is proof-to-contribution unlinkable if the advantage ${\mathrm{Adv}}_{R}^{\mathrm{pclink}}\left(A\right)$ of any probabilistic polynomial time adversary A in the following experiment is negligible:*

1. *The key generation algorithm $\left(\mathrm{sk},\mathrm{vk}\right)\leftarrow \mathrm{Kg}\left(1^{k}\right)$ is run and A is given (sk,vk).*
2. *The adversary A outputs two different serial numbers $\left(s_{0},s_{1}\right)$ sorted in lexicographic order.*
3. *A random bit b∈{0,1} is chosen.*
4. *The adversary A is allowed to play the role of the Master in the two runs of the receipt-issuing process $\sigma_{b}\leftarrow \langle A\left(\mathrm{sk},\mathrm{vk}\right),\mathrm{Helper}\left(s_{b},\mathrm{vk}\right)\rangle$ and $\sigma_{\bar{b}}\leftarrow \langle A\left(\mathrm{sk},\mathrm{vk}\right),\mathrm{Helper}\left(s_{\bar{b}},\mathrm{vk}\right)\rangle$ where $\bar{b}$ denotes the bitwise complement of b.*
5. *Two reward-requesting messages $\left(s_{0},\sigma_{0}\right)$ and $\left(s_{1},\sigma_{1}\right)$ are given to A.*
6. *A outputs a guess bit $b^{\prime }\in \left\{0,1\right\}$.*

*The adversary A succeeds if $b=b^{\prime }$. The advantage ${\mathrm{Adv}}_{R}^{\mathrm{pclink}}\left(A\right)$ is defined as $|\mathrm{Pr}\left[b=b^{\prime }\right]-\frac{1}{2}|$.*

Proof-to-contribution unlinkability requires that the adversary acting as both the *Master* and the RS should not learn any private information of the *Helper* during the receipt-issuing process. In step 2, A outputs two serial numbers $\left(s_{0},s_{1}\right)$ and in step 3, a random bit *b* is chosen. If b=0, A acting as the *Master* engages in two runs of the receipt-issuing process with the *Helper* whose serial numbers are in lexicographic order and otherwise, in reverse lexicographic order. In step 5, A acting as the RS is given two reward-requesting messages $\left(s_{0},\sigma_{0}\right)$ and $\left(s_{1},\sigma_{1}\right)$ always in lexicographic order of serial numbers (regardless of the choice of *b*). In step 6, A outputs a bit $b^{\prime }$ guessing whether b=0 or b=1. The advantage of A is defined as $|\mathrm{Pr}\left[b=b^{\prime }\right]-\frac{1}{2}|$. Note that two serial numbers $\left(s_{0},s_{1}\right)$ in step 2 must be different; if $s_{0}=s_{1}$, two runs of the receipt-issuing process in STEP 4 are independent of the choice of *b* and the whole experiment becomes meaningless.

Proof-to-contribution unlinkability ensures that any relation between a contribution and a proof cannot be identified. A reward-requesting message (s,σ) is a receipt for a randomly chosen sequence number and thus any relation between two proofs is defined by the relation between their corresponding contributions.

**Definition** **4** (Contribution-to-Contribution Unlinkability). *A reward scheme R=(Kg,Helper,Master,Vrfy) is contribution-to-contribution unlinkable if the advantage ${\mathrm{Adv}}_{R}^{\mathrm{clink}}\left(A\right)$ of any probabilistic polynomial time adversary A in the following experiment is negligible:*

1. *The key generation algorithm $\left(\mathrm{sk},\mathrm{vk}\right)\leftarrow \mathrm{Kg}\left(1^{k}\right)$ is run and A is given (sk,vk).*
2. *The adversary A outputs two pairs of serial numbers $\left(s_{\left(0,0\right)},s_{\left(0,1\right)}\right)$ and $\left(s_{\left(1,0\right)},s_{\left(1,1\right)}\right)$.*
3. *A random bit b∈{0,1} is chosen.*
4. *The adversary A is allowed to play the role of the Master in the two runs of the receipt-issuing process $\sigma_{\left(b,0\right)}\leftarrow \langle A\left(\mathrm{sk},\mathrm{vk}\right),\mathrm{Helper}\left(s_{\left(b,0\right)},\mathrm{vk}\right)\rangle$ and $\sigma_{\left(b,1\right)}$←〈A(sk,vk), $\mathrm{Helper}(s_{\left(b,1\right)},\mathrm{vk})\rangle$.*
5. *A outputs a guess bit $b^{\prime }\in \left\{0,1\right\}$.*

*The adversary A succeeds if $b=b^{\prime }$. The advantage ${\mathrm{Adv}}_{R}^{\mathrm{clink}}\left(A\right)$ is defined as $|\mathrm{Pr}\left[b=b^{\prime }\right]-\frac{1}{2}|$.*

In privacy-preserving reward schemes, the *Master* should not learn any information on the serial number that is the private input to the *Helper*. To formulate the contribution-to-contribution unlinkability, the dishonest (or curious) *Master* A is challenged to distinguish between the runs of the Helper algorithm. In step 2, A outputs two pairs of serial numbers $\left(s_{\left(0,0\right)},s_{\left(0,1\right)}\right)$ and $\left(s_{\left(1,0\right)},s_{\left(1,1\right)}\right)$. As there is no restriction on the selection of the serial numbers, A can choose two pairs as distinct as possible. For example, A may choose $s_{\left(0,0\right)}=s_{\left(0,1\right)}$ and $s_{\left(1,0\right)}=\bar{s_{\left(1,1\right)}}$ where $\bar{s}$ denotes the bitwise complement of *s*. In step 3, a random bit *b* is chosen and in step 4, A interacts with the *Helper* whose serial numbers are $\left(s_{\left(b,0\right)},s_{\left(b,1\right)}\right)$. In step 5, A outputs a bit $b^{\prime }$ guessing whether b=0 or b=1. As the probability that any random bit $b^{\prime }$ is correct (i.e., $b=b^{\prime }$) is $\frac{1}{2}$, the advantage of the adversary is defined as $|\mathrm{Pr}\left[b=b^{\prime }\right]-\frac{1}{2}|$. Contribution-to-contribution unlinkability assumes the strongest possible adversary by allowing the adversary to choose the serial numbers that are the *Helper*’s private information. This reflects the imperfectness of real-life pseudorandom number generators. Contribution-to-contribution unlinkability requires that the *Master* should not distinguish between the runs of the Helper algorithm even with known private inputs.

**Definition** **5** (Unforgeability)**.** *A reward scheme R=(Kg,Helper,Master,Vrfy) is unforgeable if for any polynomial ℓ, the advantage ${\mathrm{Adv}}_{R}^{\mathrm{forge}}\left(A\right)$ of any probabilistic polynomial time adversary A in the following experiment is negligible:*

1. *The key generation algorithm $\left(\mathrm{sk},\mathrm{vk}\right)\leftarrow \mathrm{Kg}\left(1^{k}\right)$ is run and A is given vk.*
2. *The adversary A is allowed to play the role of the Helper in the polynomially many runs of the receipt-issuing process $\sigma_{i}\leftarrow \langle \mathrm{Master}\left(\mathrm{sk},\mathrm{vk}\right),A\left(s_{i},\mathrm{vk}\right)\rangle$ for i=1,2,…,ℓ with ℓ=ℓ(k).*
3. *A outputs $\left(\left(s_{1}^{\prime },\sigma_{1}^{\prime }\right),\left(s_{2}^{\prime },\sigma_{2}^{\prime }\right),\ldots ,\left(s_{\ell +1}^{\prime },\sigma_{\ell +1}^{\prime }\right)\right)$.*

*The advantage of the adversary ${\mathrm{Adv}}_{R}^{\mathrm{forge}}\left(A\right)$ is defined as the probability that for all i=1,2,…,ℓ+1, it holds that Vrfy(vk, $s_{i}^{\prime }$, $\sigma_{i}^{\prime },L_{i})=\mathrm{valid}$ where $L_{i}$ is updated to contain all expired serial numbers, i.e., $L_{i}=L_{i-1}\cup \left\{s_{i-1}^{\prime }\right\}$ for $L_{0}=\emptyset$ (empty set) and $s_{0}^{\prime }=\epsilon$ (empty string).*

In the definition of unforgeability, the dishonest *Helper* A is required to output a forged receipt after engaging in *ℓ* receipt-issuing processes with the *Master*. As *ℓ* valid receipts are given to A during the receipt-issuing processes of STEP 2, A is required to output ℓ+1 receipts in STEP 3. Note that the ℓ+1 serial numbers in STEP 3 are not explicitly required to be all distinct and thus A can launch a double-spending attack. However, if a serial number is repeated, i.e., $s_{i}^{\prime }=s_{j}^{\prime }$ for some i<j, then $\left(s_{j}^{\prime },\sigma_{j}^{\prime }\right)$ cannot pass the verification because the list $L_{j}$ will already include $s_{i}^{\prime }$, which results in $\mathrm{Vrfy}(\mathrm{vk},s_{j}^{\prime },\sigma_{j}^{\prime },L_{j})=\mathrm{invalid}$.

### 4.2. Security Analysis

**Theorem** **1.** *The proposed reward scheme is proof-to-contribution unlinkable.*

**Proof.**  According to the proof-to-contribution unlinkability experiment, the adversary A is given (sk,vk)=(x,y) and outputs two serial numbers $\left(s_{0},s_{1}\right)$ in lexicographic order. The adversary A is allowed to play the role of the *Master* in the two runs of the receipt-issuing process $\sigma_{b}\leftarrow \langle A\left(\mathrm{sk},\mathrm{vk}\right),\mathrm{Helper}\left(s_{b},\mathrm{vk}\right)\rangle$ and $\sigma_{\bar{b}}\leftarrow \langle A\left(\mathrm{sk},\mathrm{vk}\right),\mathrm{Helper}\left(s_{\bar{b}},\mathrm{vk}\right)\rangle$ where *b* is an unknown random bit that A is challenged to guess. After the receipt-issuing process, two reward-requesting messages $\left(s_{0},\sigma_{0}\right)$ and $\left(s_{1},\sigma_{1}\right)$ are given to A. During the receipt-issuing process, A is given $h\leftarrow g^{r}H\left(s\right)$ for $s=s_{0}$ or $s_{1}$ and thus A has to find whether *h* is related to $\left(s_{0},\sigma_{0}\right)$ or $\left(s_{1},\sigma_{1}\right)$ to guess the random bit *b* correctly.

As *g* is a generator of G of prime order *p*, *H* is a full-domain hash function $H:{\left\{0,1\right\}}^{*}\to G$, and $g^{r}$ for $r\overset{R}{\leftarrow }Z_{p}$ is a uniformly random value of G, *h* is a uniformly random value of G irrespective of *s*. The independency of *h* and *s* also implies the independency of *h* and $\sigma =H{\left(s\right)}^{x}$. For any $h^{\prime }\in G$, $s^{\prime }\in S$, and $\sigma^{\prime }=H{\left(s^{\prime }\right)}^{x}$, we have

(2) $$\mathrm{Pr}\left[h=h^{\prime }\;|\;s=s^{\prime },\;\sigma =\sigma^{\prime }\right]=\mathrm{Pr}\left[h=h^{\prime }\;|\;s=s^{\prime }\right]=\mathrm{Pr}\left[h=h^{\prime }\right]=\frac{1}{\left|G\right|}=\frac{1}{\left|p\right|}$$

where the first equality follows from the fact that $s=s^{\prime }$ implies $\sigma =H{\left(s^{\prime }\right)}^{x}$. Therefore, the adversary cannot succeed in guessing the random bit *b* with non-negligible advantage; for the guess bit $b^{\prime }$ of A, the advantage $|\mathrm{Pr}\left[b=b^{\prime }\right]-\frac{1}{2}|$ is negligible. As the hash function *H* is not required to be a random oracle, the proposed reward scheme is proof-to-contribution unlinkable unconditionally. □

**Theorem** **2.** *The proposed reward scheme is contribution-to-contribution unlinkable.*

**Proof.**  According to the contribution-to-contribution unlinkability experiment of the definition, the adversary A is given (sk,vk) and outputs two pairs of serial numbers $\left(s_{\left(0,0\right)},s_{\left(0,1\right)}\right)$ and $\left(s_{\left(1,0\right)},s_{\left(1,1\right)}\right)$. The adversary A is allowed to play the role of the *Master* in the two runs of the receipt-issuing process $\sigma_{\left(b,0\right)}\leftarrow \langle A\left(\mathrm{sk},\mathrm{vk}\right),\mathrm{Helper}\left(s_{\left(b,0\right)},\mathrm{vk}\right)\rangle$ and $\sigma_{\left(b,1\right)}\leftarrow \langle A\left(\mathrm{sk},\mathrm{vk}\right),\mathrm{Helper}\left(s_{\left(b,1\right)},\mathrm{vk}\right)\rangle$ where *b* is an unknown random bit that A is challenged to guess. During the receipt-issuing process, A is given $h\leftarrow g^{r}H\left(s\right)$ for $s=s_{\left(b,0\right)}$ or $s_{\left(b,1\right)}$ and thus A has to extract some information on *s* from *h* to guess the random bit *b* correctly. However, we argue that it is impossible to obtain any information on *s* from *h* because *h* is independent of *s*.

Recall that *g* is a generator of G of prime order *p* and *H* is a full-domain hash function $H:{\left\{0,1\right\}}^{*}\to G$. As *h* is computed by $h\leftarrow g^{r}H\left(s\right)$ where $r\overset{R}{\leftarrow }Z_{p}$ is chosen uniformly at random, $g^{r}$ is a uniformly random value of G and *h* is also a uniformly random value of G irrespective of the value of H(s). For any $h^{\prime }\in G$ and $s^{\prime }\in S$, we have

(3) $$\mathrm{Pr}\left[h=h^{\prime }\;|\;s=s^{\prime }\right]=\mathrm{Pr}\left[h=h^{\prime }\right]=\frac{1}{\left|G\right|}=\frac{1}{\left|p\right|}$$

Therefore, the adversary cannot succeed in guessing the random bit *b* with non-negligible advantage; for the guess bit $b^{\prime }$ of A, the advantage $|\mathrm{Pr}\left[b=b^{\prime }\right]-\frac{1}{2}|$ is negligible. Since the hash function *H* is not required to be a random oracle, the proposed reward scheme is contribution-to-contribution unlinkable unconditionally. □

**Theorem** **3.** *The proposed reward scheme is unforgeable under the chosen-target CDH assumption in the random oracle model.*

**Proof.**  Let R=(Kg,Helper,Master,Vrfy) be the proposed reward scheme. Suppose A is a polynomial time forger algorithm against R with non-negligible advantage ${\mathrm{Adv}}_{R}^{\mathrm{forge}}\left(A\right)$ in the random oracle model. We show how to construct a polynomial time algorithm B that breaks the chosen-target CDH assumption with non-negligible probability. According to the definition of the Chosen-Target CDH Problem, B is given (p,g,y) where *g* is a generator of a cyclic group G of prime order *p* and $y=g^{x}$. Algorithm B also has access to the target oracle $T_{G}$ and the exponentiation oracle ${𝛆}_{x}$. Algorithm B simulates the attack environment of A as follows.

- **Setup:** Algorithm B starts by giving A the public parameter (p,g) and the verification key vk=y.
- ***H*-queries:** At any time, algorithm A can query the random oracle *H*. To respond to these queries, algorithm B maintains a list of pairs $\left(s_{i},w_{i}\right)$ as explained below. We refer to this list as the *H*-list. When A queries the oracle *H* at a point $s_{i}$, algorithm B responds as follows.
  1. If the query $s_{i}$ already appears on the *H*-list in a pair $\left(s_{i},w_{i}\right)$, algorithm B responds with $H\left(s_{i}\right)=w_{i}\in G$.
  2. Otherwise, B forwards $s_{i}$ to its target oracle $T_{G}$. Let $w_{i}$ be the answer of $T_{G}$ (i.e., $w_{i}=T_{G}\left(s_{i}\right)$). Algorithm B adds $\left(s_{i},w_{i}\right)$ to the *H*-list and responds to A by setting $H\left(s_{i}\right)=w_{i}$.
- **Master-queries:** As the *Master* in the receipt-issuing process has only one move, it is enough to give A access to a *Master* oracle ${\left(\cdot \right)}^{x}$, where *x* is a secret receipt-issuing key of the *Master*. Though B does not know the receipt-issuing key *x*, B can simulate the *Master* oracle by making queries to its exponentiation oracle. When A makes a query $h_{i}$ to the *Master* oracle, algorithm B forwards $h_{i}$ to its exponentiation oracle ${𝛆}_{x}$. Let $\psi_{i}$ be the answer of ${𝛆}_{x}$ (i.e., $\psi_{i}={𝛆}_{x}\left(h_{i}\right)$). Algorithm B returns $\psi_{i}$ to A.
- **Output:** Let *ℓ* be the number of the *Master* oracle queries that A has made. Eventually algorithm A produces $(\left(s_{1}^{\prime },\sigma_{1}^{\prime }\right)$, ($s_{2}^{\prime },\sigma_{2}^{\prime }$), …, $\left(s_{\ell +1}^{\prime },\sigma_{\ell +1}^{\prime })\right)$. If there is no pair on the *H*-list containing $s_{i}^{\prime }$ for 1≤i≤ℓ+1, then B makes a query itself for $H(s_{i}^{\prime })$ to ensure that such a pair exists. For each 1≤i≤ℓ+1, algorithm B finds $\left(s_{i}^{\prime },w_{j_{i}}\right)$ in the *H*-list and outputs $\left(\left(\sigma_{1}^{\prime },j_{1}\right),\left(\sigma_{2}^{\prime },j_{2}\right),\ldots ,\left(\sigma_{\ell +1}^{\prime },j_{\ell +1}\right)\right)$.

Let $q_{T}$ be the number of queries *B* made to the target oracle $T_{G}$. As algorithm B makes a target oracle query only if A makes a *Master* oracle query, we have $q_{T}=\ell$. It is easy to see that the view of A in the simulated experiment is indistinguishable from its view in the real experiment and that B is successful whenever A is successful. Therefore, the polynomial time algorithm B can break the chosen-target CDH assumption with non-negligible advantage ${\mathrm{Adv}}_{G}^{\mathrm{ct}-\mathrm{cdh}}\left(B\right)={\mathrm{Adv}}_{R}^{\mathrm{forge}}\left(A\right)$. □

Finally, we show that our scheme satisfies the security property P3 (non-repudiation) as follows. Because the proof σ is signed by the *Master* (see definition 3), RS can verify σ with the public key *y* from the *Master*’s certificate by checking whether e(σ,g)=e(H(s),y) or not. Therefore, the *Helper* can prove that the contribution is real, and RS cannot repudiate the proof. The system guarantees non-repudiation.

### 4.3. Implementation & Evaluation

#### 4.3.1. Implementation Details

We made the implementation in Java 8. The RS is implemented using spring boot [56] with version 2.0.0. The *Master* and the *Helper* are implemented in Android version 8 and 9. For the key generation and signatures we use the “Java Pairing-Based Cryptography Library” [57] version 2.0.0, and for the certificate related functions we use the “Bouncy Castle” [58] with version 1.62. For the serialization of the data during communication, we use “Protocol Buffers” [59] with version 3.0.0.

#### 4.3.2. Experiment Methods

##### Experiment Environments

For experiment, as shown in Table 1, we used one computer and two Android devices, one of them is Samsung Galaxy J7 with Android version 8, another Android device is Samsung Galaxy S8 Android version 9, the computer has CPU with clock speed of 3.4 GHz. We tested the receipt issue process between the *Helper* and the *Master* using the two Androiddevices. The *Helper* runs on Android device with version 9, and the *Master* runs on Android device with version 8. The reward process between the *Helper* and the RS is tested between the computer with Windows 7 64-bit and the Android device with version 9.

##### Metric

We have two metrics to evaluate our system: The first one is the measurement of the system latency. The second metric is the analysis of the storage usage in the *Helper* for receipts. To measure the latency of our system, we calculate the average latency of each steps related to the operation like generation, signing, and verification. For the accuracy of the communication latency, we measure the total time from the packet sending to the packet receiving only from the *Helper* side. In our system the receipts can be aggregated, the storage usage of the receipts with and without aggregation are different. We calculate the storage usage using various numbers of the receipts.

#### 4.3.3. Experiment Results

In our implementation, we are assuming that there is only one *Master*. For the aggregated receipts, we only verify one certificate, because all of the receipts come from the same *Master*. The verification function is compatible with more than one certificate from different *Masters*. All the processes are tested more than 20 times, especially the experiments for issue of the receipt over 3000 times. To compare the difference between reward claim with aggregated receipts and without aggregated receipts, we made tests for five different numbers of aggregated receipts. The storage in the *Helper* needed for aggregated receipts and receipts without aggregation are manually calculated.

Table 2 shows the total latency for issue of one receipt and the claim of the reward using one receipt. The table consists of six columns. The first column indicates the process, and the second column indicates the actor, which will run the step in the third column. The next column is the average latency for the steps. The fifth column is the average communication latency including the time from the packet sending to the packet receiving. The last column is the average total latency of the process. The step 0 is one step from the setup process. We do not consider the latency of setup as the part of the total system latency.

The steps from 1 to 14 are the protocol between the *Helper* and the *Master*. The steps from 1 to 6 are for the authentication of the *Master*. We measured that the whole time being used for the authentication including communication latency is ~1.04 s. Steps from 8 to 14 are for the issue of one receipt, and the whole time being used is about 0.7 s. There are three verification steps in the table, the time they used are all about 0.2 s. Step 3 and 11 are all for signing. The step 3 is much slower than the step 11. The step 3 contains the generation of the hash for the challenge, the step 11 does not need to generate hash, because the step 9 already prepared h to be signed. Additionally, the device for the *Master* had less computation power than the device for the *Helper*. The communication latency during the authentication is about 0.13 s (step 2 plus 4) and during the issue of the receipt is about 0.14 s (step 10 plus 12).

The steps from 15 to 18 are for the reward. The time being used to claim reward for one receipt including the communication time is about 0.16 s. The communication does not cause so much latency by 0.08 s (step 15 plus 18). The total time for the *Reward Service* to verify one receipt is about 0.083 s.

Note that the delay for authentication (approximately one second) and receipt-issuing (~0.7 s) will multiply with the number of *Helpers* that participate a task of a *Master* simultaneously. However, this delay does not intervene the tasking performance between *Master* and multiple *Helpers* because the authentication step strictly occurs before any task works, and the receipt-issuance occurs after the task work. For instance, with three *Helpers*, the authentication will take about 3 s and the final receipt issuance will take about 2 s. The tasking performance of *Helpers* will be free from this delay.

**Figure 4:**
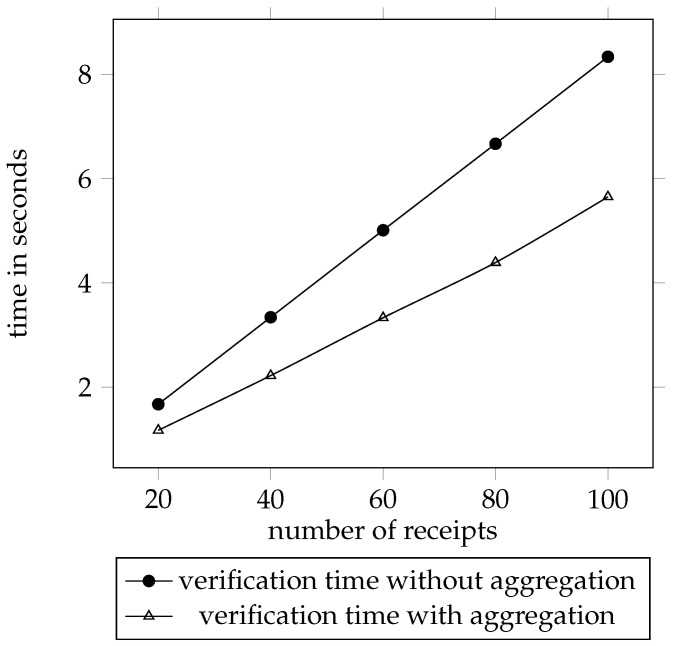
Receipt verification latency according to aggregation.

Figure 4 is the time used to verify a number of receipts with aggregation and without aggregation. The *y*-axis is the time being used to verify in seconds, and the *x*-axis is the number of receipts being tested. The line marked with circle shows the data for the verification without aggregation. The other line marked with triangle shows the data for the verification with aggregation. We tested the aggregated receipts with five different amounts and calculated the verification time of the receipt without aggregation according to an average verification time for one receipt. The line marked with circle starts from 1.67 s by 20 receipts and reaches 8.34 s by 100 receipts. The other line begins with 1.17 s by 20 aggregated receipts and reaches 5.65 s by 100 aggregated receipts.

It can be seen that the time needed for verifying receipts without aggregation is more than the time needed for verifying aggregated receipts. The reason is that, the verification of the aggregated receipts only has one σ to verify, the verification of receipts without aggregation has multiple σs to verify. We find out that, the verification time has linear relationship with the number of the receipts *n*. If we define the verification time is n∗α, the α is 0.083 for receipt verification without aggregation and it is 0.056 for receipt verification with aggregation. The distance between the two lines is getting larger along with the growing number of receipts. The time needed for verifying the aggregated receipts is not a constant on account of the growing number of the serial numbers and the certificates.

**Figure 5:**
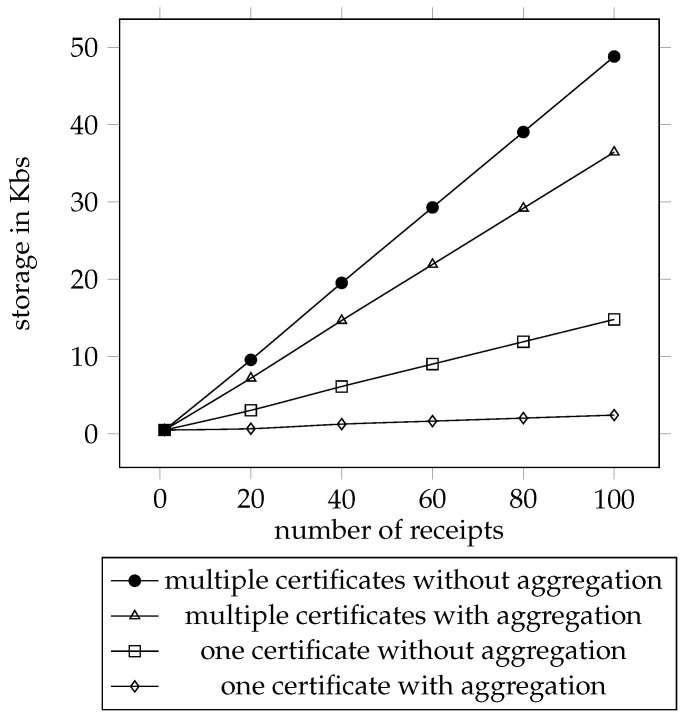
Receipt storage requirement.

Figure 5 shows the storage needed by the *Helper* for the receipts without aggregation and the aggregated receipts. The *y*-axis is the storage required in Kilobyte. The *x*-axis is the number of the receipts. As the same as the previous figure, the line on the top marked with circle is the data from the receipts without aggregation, and the second line marked with triangle is the data from the aggregated receipts. The third line marked with square is the data from the receipts without aggregation using only one certificate. The last line marked with rhombus is the data from the aggregated receipts using only one certificate. In our implementation, to store the receipt, we need to store the σ, the serial number, and the certificate. The σ requires 128 bytes, the serial number requires 20 bytes, and the certificate requires 352 bytes. According to the space requirement from the σ, the serial number, and the certificate, we calculated the storage needed to store the receipts with different number of receipts. If we define the storage used by σ as $\sigma_{S}$, the storage used by serial number as $s_{S}$, the storage used by certificate as $cert_{S}$, the amount of serial number as *i*, the amount of the certificates as *j*. The storage usage for one receipt is $\sigma_{S}+s_{S}+cert_{S}$, and the storage used for aggregated receipts is $\sigma_{S}+i*s_{S}+j*cert_{S}$

For only one receipt, the storage needed from all the four lines are the same; it is 0.49 Kbs. The storage needed for 20 receipts from the first line is 9.57 Kbs, from the second line is 7.20 Kbs, from the third line is 3.039 Kbs, and from the last line is 0.66 Kbs. All the storage requirements from the four lines grows with the number of receipts. The storage needed for 100 receipts from the first line is 48.83 Kbs, from the second line is 36.45 Kbs, from the third line is 14.8 Kbs, and from the last line is 2.42 Kbs. As the aggregated receipt has only one σ to store, the storage requirement from the second line is less than the first line. When the certificates from all the aggregated receipts are the same, only one certificate is needed to be stored, thence the storage requirement from the third and fourth line much less than the other two.

## 5. Discussion and Future Work

For the sake of privacy, all the receipts are indistinguishable. However, not all contributions are worth the same amount of rewards. To recognize the differing value of contributions, the *Master* can issue multiple receipts to the *Helper* proportional to the reward amount that the *Helper* deserves. This does not compromise the *Helper*’s privacy, because the *Helper* will claim each receipt independently and because of contribution-to-contribution unlinkability (Theorem 2) the attacker cannot identify the *Helper* by comparing with the number of receipts that the *Helper* received for its contribution. Moreover, aggregation of receipts in an arbitrary manner can also make such an attack infeasible.

**P5: Proof-to-Master unlinkability (G1)** Difficult to identify the *Master* that provided the task, which corresponds to a proof.

For the business case, usually the RS pays the rewards to the *Helper* on behalf of the *Master*. Therefore, the RS needs to know the identity of *Master*. By colluding *Master* and RS, the tasks can be linked to the proofs. It is hard to provide Proof-to-Master unlinkability. One solution is to provide the anonymity to the *Masters* using Short Group Signature from Boneh et al. [60]. The *Masters* in system will use one group public key, so that the RS can authenticate the *Master* without knowing precisely which *Master* it is. How to apply the group public key into the system is out of this paper’s scope.

Our scheme can be rewritten to employ an asymmetric bilinear map $e:G_{1}\times G_{2}\to G_{T}$ with a full-domain hash function $H:{\left\{0,1\right\}}^{*}\to G_{1}$ and a generator $g_{2}$ of $G_{2}$. The receipt $\sigma =H{\left(s\right)}^{x}$ will be an element of $G_{1}$, while the verification key $y=g_{2}^{x}$ will be an element of $G_{2}$. This setting allows us to adopt elliptic curves due to Barreto and Naehrig [61] where elements of $G_{1}$ have a 160-bit representation at the 1024-bit RSA security level, i.e., each receipt is only 160 bits.

As a reward-requesting message (or a proof) includes the serial number *s*, any information that can be deduced from serial numbers cannot be hidden. For example, if a *Helper* always uses serial numbers that are prime, proofs belonging to the *Helper* can be identified with high probability. Therefore, proof-to-proof unlinkability can be achieved only if sequence numbers are chosen uniformly at random.

In our work, the *Helper* only authenticates the *Master* at the beginning of the protocol. The communication between the *Helper* and the *Master* should be done in secure channel. There are researches about the pairing based secure channel such as the “Milagro TLS” [62] and “Secret handshakes from pairing-based key agreements” [63]. Both of them are using identity based shared secret. In the future we plan to make a pairing based secure channel with anonymity, as in our work the identity of the *Helper* is not revealed.

## 6. Related Works

Many works have proposed privacy-preserving mechanisms for incentive crowdsensing systems using various techniques. Some prior works use encryption to protect the participants’ privacy in incentive crowdsensing systems.

One of them is an auction-based incentive privacy-preserving system [40]. This system consists of the participants, crowdsensing platform, the requester, and the auction issuer. In this system, the requester submits the task to the sensing platform, which publishes tasks as auctions. Participants can interact with the auction issuer and prepare encrypted sensing profiles with the public key from the auction issuer. Participants bid the auctions while submitting their encrypted sensing profiles as bids information to the platform. The platform receives encrypted sensing profiles and chooses the winners, then signs and sends the receipts to the winners. In this work, the bids information and payment information are encrypted. However, the platform knows all the payments and will publish the identity of the winners, and thus the attacker can get the information from the platform.

In [41], Zhang et al. attempt to protect participants’ privacy when the participants send sensing data to the server. In their mobile crowdsensing system, the participants iteratively pass their data, which are tagged with their accurate locations through nearby participants to the server. The participants can upload their incentive requirement to the application server. According to the incentive budgets of tasks, the application server sends data requirement of the task to the potential participants. The server selects participants iteratively within many rounds for the task, and at each iteration the winner gets a part of requirement from the task. The previous round winner is called the next round winner’s parent. To protect the participant’s privacy, the server will randomly shuffle the array of the selected winners and the participants will use new IP addresses to send data. One aggregated report set from participants will be sent to the server. The server cannot link reward to the contribution. However, the server distributes the rewards and can use rewards information to link the contributions. The system server can collude with participants to infer the private information from the other participants. Moreover, this work does not mention the non-repudiation property of the system.

Digital currency called E-cent [42] was proposed for building an untraceable system that protects the anonymity of the participants. In their system, the participant can get task from the application server and the corresponding rewards. All the participants use different pseudonyms in a mix zone and they can change pseudonyms every time. To bootstrap, the server generates E-cents with its signature. A participant can mark E-cents with a secret number and submits a report with a pledge corresponding to a task using E-cent. As the E-cents are marked with a different secret number every time, the server cannot link the participant to the E-cents. The application pays the participant according to the contribution and thus can link the contribution to the reward. Because an attacker can use the server to make tracing attacks by randomly matching the exchange pair, the anonymity level of a participant in this work depends on the number of on-line participants in the mix zone. The more participants in mix zone collude, the easier a tracing attack can get on.

Some researchers use group signature to make privacy preserving systems. Gisdakis et al. [35] proposed a system for protecting user privacy while providing accountability for mobile crowdsensing. In this work, the participant can get rewards using a pseudonym without revealing their identity. To protect private information in the participant’s data such as health condition or context information, the participant signs a task using a group signature. If the participant wants to get a task, the Pseudonym Certification Authority provides a temporary pseudonym, so that the participant can apply the task anonymously. Therefore, the system cannot link the sensing data to participant nor the sensing data to another. However, it is still possible to break anonymity of the participant by colluding.

Similarly, Li, et al. in [43] proposed CreditCoin for vehicular announcement network using ring signature. This system is based on a blockchain and motivates the user to share traffic information with others by getting some rewards. Their system has many entities: the participant, trace manager, trusted authority, application server, consensus server, and public role. The participant can request traffic information from the application server by paying rewards and can also share traffic information corresponding to a task to get rewards. To achieve anonymity of the participants, a message should be signed by at least k participants. If a participant wants to share a message with another vehicle, k−1 other participants are invited to sign the message. Aggregated packets are used, so that the contribution and reward cannot be linked. Therefore, the participant’s identity is not revealed. However, this system requires a trusted authority to manage addresses of participants and records the relationship between addresses and participants. It compromises privacy and traceability. A trace manager can trace the malicious user, who makes fraudulent transaction, so the service quality will be ensured. Because of the traceability, an attacker colluding with the authority can trace a participant.

Besides encryption, participant coordination, group signature, and ring signature, there are also researchers choosing blind signature to protect the private information [44,45,64,65,66,67,68], because blind signature can provide unlinkability, intractability, unforgeability, and blindness. Blind signature enables the signer to sign on the message without knowing the content in the message from the user, so that the user cannot be linked to the message [69,70,71,72,73].

Wu et al., in [74], proposed a similar system to that in [43], their system can be applied not only to the vehicles, but also for the mobile phones. Different from the work in [43], they use group signature and (partial) blind signature to protect the private information and the system consists of data collectors, sensing servers, participants, group manager, and trusted pseudonym authority. In their system, the task ID and receipts will be blinded, so the group manager can neither link the task to the participant nor the receipts. The colluding between the sensing server and the group manager is not prevented.

Li and Cao [44,45] proposed credit-based privacy-preserving systems for mobile sensing using blind signature against linking attack. In their systems, the participants get tasks from the data collector and receive rewards for their sensed data. The participants register the tokens using their real identity and generate credit token identifiers. The data collector signs partially blind signature on the credit token identifiers to allow the participants to use corresponding credit tokens for one task. These credit tokens are bound to the participant’s identity. Once the data collector accepts the participants’ reports, the participant generates the report receipt token identifiers with related task and reports information and gets partially blind signature on them. The participant generates the receipts using random blind factor. Data collector gets receipts and returns pseudo-credits to the participant. The participant can transform the pseudo-credits into credit tokens by removing the blind factor. Later the participant uses his real identity to deposit the credit token.

During the processes, the data collector does not know the participant’s identity, and the blind signatures are applied to all tokens. Therefore, a receipt cannot be linked to the report even if the attacker tries to collude with the data collector. However, the data collector manages the participants’ credits accounts, so that it is possible to link a reward to a given task. As the signature from the data collector is needed in all processes, the issue of tasks and the rewards payment cannot be separated in this work. There are many collaborations between the participant and the data collector, and this brings a burden for the communication.

Dimitriou [68] made a privacy-preserving mechanism for incentive mobile crowdsensing systems using an anonymous token, zero-knowledge proof, and partially blind signature. This work provides a safe rewarding mechanism to help the system to attract more participants. Their system consists of the client and the application server. The participant communicates with the application server using an anonymous channel and manages only a single token, which can be updated with new rewards. The participant generates the secret ID using a random number. This secret ID is only known to the participant. The public ID will be generated based on the secret ID and sent to the application server. In this way the participant is registered.

The participant generates another random number as token ID and creates Pedersen commitment using his secret ID. The token ID, commitment, and a zero-knowledge proof will be sent to the application server. The application server verifies the proof and signs blindly on the token ID and commitment. The participant receives the signature on token values and collects rewards with the token. Multiple rewards can be aggregated to a single token by renewing the token ID and the commitment. When the participant wants to redeem the rewards, the public ID and the token along with the signature on its value will be sent to the application server. The application server stores the token ID in database, so that the token ID cannot be used again. For the next token, the participant uses a new token ID. In their work, rewards are not linkable to the same participant, and the partially blind signature prevents the system from the colluding attack. To achieve their goal, multiple techniques are applied, and it is not easy to implement the system. Furthermore, this work does not consider separating the reward issuer and redeem server. In some business cases the reward issuer and redeem server are not the same.

In our work, we attempt to protect the system against linking attack and colluding attack only by using blind signature, and also consider separating the reward issuer and the reward redeem server. The comparison of described works for privacy-preserving properties is shown in Table 3.

In addition, data handling techniques (e.g., data obfuscation, differential privacy) can also be used to protect the user’s privacy. For example, in [75] the researchers made a rewarding system based on anonymous vouchers. The authors use partial data disclosure and obfuscation techniques to ensure the user’s privacy. In our work, we don’t modify the data.

## 7. Conclusions

In this paper, we proposed a privacy-preserving reward system using blind signature. We defined the proof-to-contribution linking attack and the contribution-to-contribution linking attack. In our system, the user could get aggregated rewards. We proved that our system is unforgeable, contribution-to-contribution unlinkable, and proof-to-contribution unlinkable. The implementation was complete and tested in mobile devices. The experiment results show that our system is feasible and efficient. We discussed further issues of our design and details of implementation.

## Figures and Tables

**Table 1:** Experiment environment.

Actor	HW	SW
RS	Intel(R) Core(TM) i7-4770 CPU @ 3.40 GHz	Windows 7 64-bit
*Master*	Samsung Galaxy J7	Android version 8
*Helper*	Samsung Galaxy S8	Android version 9

**Table 2:** Table of latency.

	Actor	Step	Avg. Latency	Avg. Total
Setup	*Master*	0. keypair generation	0.10153 s	0.10153 s
Authentication	*Helper*	1. challenge generation	0.00061 s	1.04278 s
	*Helper*	2. request certificate and send challenge	0.06436 s	
	*Master*	3. check challenge and sign on challenge	0.41612 s	
	*Master*	4. send certificate and signed challenge	0.06436 s	
	*Helper*	5. certificate verification	0.25776 s	
	*Helper*	6. signature on challenge verification	0.23957 s	
Receipt issue	*Helper*	7. send contribution		0.70426 s
	*Helper*	8. r and s generation	0.00085 s	
	*Helper*	9. h generation	0.17215 s	
	*Helper*	10. send h	0.06770 s	
	*Master*	11. sign on h	0.12260 s	
	*Master*	12. send signed h	0.06770 s	
	*Helper*	13. receipt unpack	0.05778 s	
	*Helper*	14. receipt verification	0.21548 s	
Reward claim	*Helper*	15. send s, sigma and certificate	0.04001 s	0.16333 s
	RS	16. certificate verification	0.00805 s	
	RS	17. receipt verification	0.07526 s	
	RS	18. send reward	0.04001 s	

**Table 3:** Comparison of related works for privacy-preserving properties.

	Non-Repudiation	Proof-to-Contribution Unlinkability	Contribution-to-Contribution Unlinkability	Against Colluding
Sun [40]	Yes	No	No	No
Zhang, et al. [41]	No	Yes	No	No
Niu, et al. [42]	Yes	No	No	No
Gisdakis, et al. [35]	Yes	Yes	Yes	No
Li, et al. [43]	Yes	Yes	Yes	No
Wu, et al. [74]	Yes	Yes	Yes	No
Li and Cao [44,45]	Yes	Yes	No	Yes
Dimitriou [68]	Yes	Yes	Yes	Yes
PARS (this work)	Yes	Yes	Yes	Yes

## Data Availability

Not applicable.
